# Redox Regulation of Signaling Complex between Caveolin-1 and Neuronal Calcium Sensor Recoverin

**DOI:** 10.3390/biom12111698

**Published:** 2022-11-16

**Authors:** Vasiliy I. Vladimirov, Margarita P. Shchannikova, Alexey V. Baldin, Alexey S. Kazakov, Marina P. Shevelyova, Aliya A. Nazipova, Viktoriia E. Baksheeva, Ekaterina L. Nemashkalova, Anastasia S. Frolova, Natalia K. Tikhomirova, Pavel P. Philippov, Andrey A. Zamyatnin, Sergei E. Permyakov, Dmitry V. Zinchenko, Evgeni Yu. Zernii

**Affiliations:** 1 Shemyakin and Ovchinnikov Institute of Bioorganic Chemistry, Russian Academy of Sciences, Moscow 117997, Russia; 2 Branch of Shemyakin and Ovchinnikov Institute of Bioorganic Chemistry, Russian Academy of Sciences, Pushchino 142290, Russia; 3 Belozersky Institute of Physico-Chemical Biology, Lomonosov Moscow State University, Moscow 119992, Russia; 4 Institute for Biological Instrumentation, Pushchino Scientific Center for Biological Research of the Russian Academy of Sciences, Pushchino 142290, Russia; 5 Institute of Molecular Medicine, Sechenov First Moscow State Medical University, Moscow 119991, Russia; 6 Scientific Center for Translation Medicine, Sirius University of Science and Technology, Sochi 354340, Russia

**Keywords:** retina, photoreceptor, oxidative stress, apoptosis, caveolin-1, recoverin

## Abstract

Caveolin-1 is a cholesterol-binding scaffold protein, which is localized in detergent-resistant membrane (DRM) rafts and interacts with components of signal transduction systems, including visual cascade. Among these components are neuronal calcium sensors (NCSs), some of which are redox-sensitive proteins that respond to calcium signals by modulating the activity of multiple intracellular targets. Here, we report that the formation of the caveolin-1 complex with recoverin, a photoreceptor NCS serving as the membrane-binding regulator of rhodopsin kinase (GRK1), is a redox-dependent process. Biochemical and biophysical in vitro experiments revealed a two-fold decreased affinity of recoverin to caveolin-1 mutant Y14E mimicking its oxidative stress-induced phosphorylation of the scaffold protein. At the same time, wild-type caveolin-1 demonstrated a 5–10-fold increased affinity to disulfide dimer of recoverin (dRec) or its thiol oxidation mimicking the C39D mutant. The formation of dRec in vitro was not affected by caveolin-1 but was significantly potentiated by zinc, the well-known mediator of redox homeostasis. In the MDCK cell model, oxidative stress indeed triggered Y14 phosphorylation of caveolin-1 and disulfide dimerization of recoverin. Notably, oxidative conditions promoted the accumulation of phosphorylated caveolin-1 in the plasma membrane and the recruitment of recoverin to the same sites. Co-localization of these proteins was preserved upon depletion of intracellular calcium, i.e., under conditions reducing membrane affinity of recoverin but favoring its interaction with caveolin-1. Taken together, these data suggest redox regulation of the signaling complex between recoverin and caveolin-1. During oxidative stress, the high-affinity interaction of thiol-oxidized recoverin with caveolin-1/DRMs may disturb the light-induced translocation of the former within photoreceptors and affect rhodopsin desensitization.

## 1. Introduction

Caveolin-1 is a 22 kDa transmembrane scaffold protein (178 amino acids), which is expressed in various cell types throughout the organism and involved in lipid metabolism, protein–membrane targeting, endocytosis, mechanotransduction, and cell signaling [1,2,3]. Being localized mainly in detergent-resistant membrane (DRM) rafts, it assembles into oligomeric complexes and with the assistance of cavin family proteins induces plasma membrane curvature thereby promoting biogenesis of caveolae, membrane invaginations accommodating components of signal transduction systems [4,5,6]. In DRMs, caveolin-1 binds and regulates multiple signaling proteins, including receptors (EGFR, TGFBR, mGluR1, IP3R1), alpha-subunits of G-proteins, various effector enzymes (adenylyl cyclase, protein kinase C, phospholipase D, NO-synthetase), signal transducer proteins (β-catenin, dipeptidyl peptidase-4), and others (for review, see [2,7]). The simultaneous association of different signaling proteins with caveolin-1 provides their compartmentalization in caveolae, thereby increasing the efficacy of signal transduction. Furthermore, caveolin-1 can directly affect signaling proteins’ activity by regulating them negatively and positively [2]. Given its properties, caveolin-1 plays a role in regulating cell migration, proliferation, and apoptosis, and has been linked to various pathologies, such as diabetes, cancer, cardiovascular disease, atherosclerosis, kidney disease, degenerative muscular dystrophies, as well as age-related macular dystrophy (AMD) and glaucoma [1,5].

**Figure 1:**
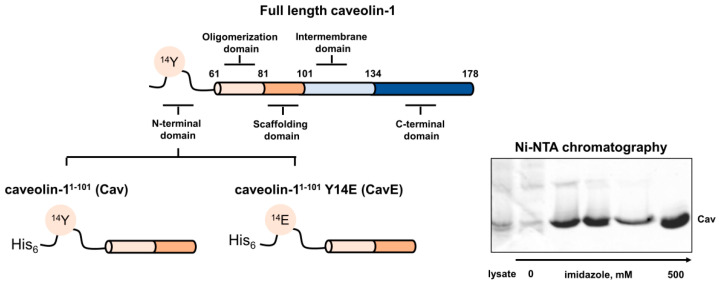
Schematic representation of domain organization of caveolin-1 and structure of its fragments, Cav and CavE, prepared in this study. SDS-PAGE represents fractions obtained during purification of Cav using Ni-NTA affinity chromatography.

The membrane-sculpting function of caveolin-1 and its scaffolding activity towards signal transduction components and other proteins are provided by the specific structure of the protein, where the central hydrophobic part (102–134 aa) is inserted into the membrane, whereas the N-terminus (1–101 aa) and C-terminus (135–178 aa) face the cytoplasm. Four functional domains of caveolin-1 are conventionally distinguished: the N-terminal domain (1–81 aa), oligomerization domain (OD, 61–101 aa), intramembrane domain (ID; 102–134 aa), and C-terminal domain (135–178 aa) [8,9] (Figure 1). A recent cryo-EM study suggested an overall architecture of the 8S caveolin-1 complex, in which 11 protein molecules are shaped in a membrane-embedded disc [4]. In each protomer, ID is folded into two α-helices, which penetrate the membrane. The C-terminal domain consists of an amphipathic α-helical “spoke region” (SR, 135–169 aa) and a β-strand (170–176 aa). It contains ubiquitination sites facing the cytoplasm and palmitoyl groups (at C133, C143, and C156) anchoring the C-terminus to the membrane. OD is generally recognized as a motif mediating the formation of caveolin-1 oligomers, which is important for the organization of the caveolae [8]. Indeed, in the 8S caveolin-1 complex, the protomers contact mostly via the residues from OD, although the complex is stabilized by several additional interactions involving amino acids from the N-terminus and ID [4]. The part of OD called the “scaffold domain” (SD; 82–101 aa) represents the main functional unit of the protein. It is enriched in aromatic residues and recognizes signaling targets containing the caveolin-binding motif (CBM). SD is localized close to the phospholipid bilayer but is sufficiently exposed to the cytoplasm to support protein–protein interactions [4,10]. The data regarding the secondary structure of SD are contradictory. Thus, NMR studies report that the soluble peptide corresponding to this domain (83–102 aa) forms a β-strand [10]. Meanwhile, in the NMR structure of a large C-terminal fragment of caveolin-1 (62–178 aa) embedded in lyso-myristoylphosphatidylglycerol micelles and the cryo-EM structure of the full-size protein expressed in bacteria SD comprises a single α-helix [4,11]. The N-terminal domain of caveolin-1 (the tertiary structure of this part of the protein is not yet completely resolved [4]) seems to play a regulatory function, as it contains a phosphorylation site for Src tyrosine kinases at Y14 and an additional phosphorylation site at S80. The first site is involved in the regulation of the signaling activity of caveolin-1, whereas the second site participates in its secretion [9,12].

Caveolin-1 is abundantly expressed in the retina, where it is found in vascular cells, Müller glia, retinal pigment epithelium (RPE), and photoreceptors [5]. Accordingly, it was implicated in retinal development, maintaining the blood–retinal barrier by retinal vascular endothelial cells, as well as the phagosomal function of RPE. The role of caveolin-1 in mature photoreceptors remains less clear even though it was demonstrated to interact with visual cascade proteins (i.e., transducin) in vitro, and its genetic ablation induced significant photoresponse deficits [5,13]. Previously, we have suggested that the activity of caveolin-1 in photoreceptor cells can be related to its interaction with neuronal calcium sensors (NCSs), myristoylated membrane-binding proteins involved in Ca^2+^-dependent regulation of phototransduction by modulating the activity of visual cascade enzymes. Indeed, caveolin-1 can bind photoreceptor proteins of the NCS family, including NCS-1, recoverin, guanylate cyclase-activating protein-1 (GCAP1), and GCAP2. All of them were co-fractionated with caveolin-1 in DRMs, isolated from illuminated bovine rod outer segments, and exhibited high-affinity interaction with CSD-containing fragments of caveolin-1 in the absence of calcium [14].

Among the retinal NCS proteins, the most evidenced interaction with caveolin-1 was observed in the case of recoverin. Early work demonstrated co-immunoprecipitation of these proteins from cellular extracts [15], while their direct interaction under Ca^2+^-free conditions was confirmed in subsequent studies [14,16]. Furthermore, recoverin was demonstrated to contain a specific binding site for caveolin-1, composed of aromatic amino acids localized in the C-terminal domain of the protein [14]. Being a photoreceptor-specific protein, recoverin binds to membranes of photoreceptor outer segments (OS) via the myristoyl group and regulates the desensitization of rhodopsin by rhodopsin kinase (G-protein coupled receptor kinase-1, GRK1) in a Ca^2+^-dependent manner [17,18]. An important feature of recoverin is its light-induced translocation from OS to inner segments (IS) of photoreceptors, with a small fraction of the protein remaining in the illuminated OS (at low calcium levels) [19]. It was suggested that this residual Ca^2+^-free recoverin is retained in DRMs due to its interaction with caveolin-1. Moreover, such protein is characterized by increased affinity to calcium, which would enable GRK1 inhibition at background light level [14,16]. The cooperative action of recoverin and caveolin-1 in the retina is supported by the electrophysiological studies, demonstrating similarly diminished flash sensitivity at background light level in rods of Rec-/- and Cav1-/- mice [13,20].

Notably, the retina is highly vulnerable to oxidative stress. Indeed, the high oxygen consumption combined with constant exposure to the light and the abundance of photosensitizer molecules induces multiple photochemical reactions in RPE and photoreceptors leading to the formation and accumulation of the reactive oxygen species (ROS). The inability of the intrinsic antioxidant defense to neutralize ROS, especially in the elderly, can cause apoptotic death of these cells, the major pathogenic factor of AMD, a common cause of blindness [21,22,23]. Along with oxidative stress, AMD progression is enhanced by a deficiency of zinc. A healthy retina contains a high concentration of zinc, which performs multiple functions, including modifying photoreceptor membranes, regulating visual cascade proteins, modulating synaptic transmission, and playing a structural role in retinal antioxidant enzymes [24,25]. Under normal conditions, zinc exists mainly in high-affinity complexes with proteins (mostly, cysteine-rich metallothioneins), whereas oxidative stress leads to their oxidation and the release of the metal, which can be captured by multiple low-affinity sites. Light-induced oxidative stress and increased concentration of such “mobile” zinc represent powerful cytotoxic factors for retinal cells along with zinc deficiency [26,27]. Growing evidence indicates that both caveolin-1 and recoverin can respond to oxidative and zinc-dependent conditions in the retinal photoreceptors. Thus, oxidative stress induces phosphorylation of caveolin-1 at Y14 [28,29,30], and the phosphorylated form of the protein was found to accumulate in OS [31,32]. In turn, recoverin responds to light-induced oxidative stress of photoreceptors by thiol oxidation of its single cysteine-39 in a Ca^2+^-dependent manner: in the presence of calcium, it forms disulfide dimer (dRec), whereas in the absence of calcium it forms oxidized monomer containing sulfenic/sulfinic/sulfonic group [33,34,35,36]. Both modifications highly affect the signaling functions of caveolin-1 and recoverin, which makes them redox-sensitive proteins [34,35,36,37]. Moreover, our recent studies indicate that NCSs, including recoverin, can bind zinc and the binding affects their non-covalent and disulfide (covalent) dimerization, thereby contributing to the redox regulation of these proteins [38,39,40].

Taking these findings into account, in the current work we focused on the impact of oxidative stress conditions on the signaling complex between caveolin-1 and recoverin. Using biochemical and biophysical techniques, we examined the effect of redox-dependent modifications of caveolin-1 and recoverin on their interaction in vitro. Furthermore, we monitored the interaction between these proteins under physiological conditions in an appropriate cellular model. Our results point to the redox sensitivity of the recoverin–caveolin-1 complex and provide novel insights into the mechanism of adaptation of the visual system to oxidative conditions.

## 2. Materials and Methods

### 2.1. Materials

All reagents and kits for plasmid construction were from Evrogen (Moscow, Russia) or Promega (Madison, WI, USA). Chromatography resins and pre-packed columns were from GE Lifesciences (Chicago, IL, USA). Reagents and consumables for Western blotting and surface plasmon resonance spectroscopy were from Bio-Rad (Hercules, CA, USA). Culture media and reagents for cellular biology were from Gibco (Amarillo, TX, USA), Thermo Fisher (Waltham, MA, USA), and PAN Biotech (Aidenbach, Germany). Rabbit monoclonal antibodies against caveolin-1 and phosphorylated caveolin-1 and mouse monoclonal antibodies against recoverin were from Santa Cruz Biotechnology (Dallas, TX, USA). Secondary antibodies conjugated with peroxidase were from Jackson Immunoresearch (West Grove, PA, USA). TurboFect transfection reagent, bicinchoninic acid (BCA) Protein Assay Kit, goat anti-rabbit Alexa Fluor 555-conjugated IgG, goat anti-mouse Alexa Fluor 488-conjugated IgG, and 4′,6-diamidino-2-phenylindole (DAPI) were from Thermo Fisher (Waltham, MA, USA). Equipment and reagents for microscopy were from Zeiss (Oberkochen, Germany). Ionomycin, auranofin, EDTA-free protease inhibitor cocktail, and Ellmann’s reagent (5,5′-dithiobis (2-nitrobenzoic) acid) were from Sigma-Aldrich (St. Louis, MO, USA). Other chemicals were from Sigma-Aldrich, AppliChem (Darmstadt, Germany), or Amresco (Dallas, TX, USA) and were at least of reagent grade.

### 2.2. Preparation of Caveolin-1 Forms

The genetic construct encoding the N-terminal fragment of caveolin-1 M1-R101 was obtained previously [14]. The respective DNA fragment was sub-cloned into the pET-22b vector to ensure expression of the protein with N-terminal His6-tag (Cav). The N-terminally tagged mutant CavE was constructed on the base of Cav by introducing Y14E substitution using standard site-directed mutagenesis techniques. Both recombinant proteins were produced in Escherichia coli strain BL21 Star TM (DE3). The bacteria were grown in 2YT medium at 37 °C with shaking (200 rpm) until A_595_ = 1.0. The expression of the target genes was induced by the addition of 0.5 mM IPTG. The cells were cultivated for 6 h, harvested by centrifugation (3000× *g*), and the pellet was suspended in lysis buffer (50 mM Tris-HCl buffer, pH 7.5, 1 mM EDTA, 5 mM MgCl_2_, 5 mM 2-mercaptoethanol, 25 mM sucrose), containing 50 μg/mL lysozyme, incubated for 20 min and frozen at −20 °C. After thawing, the suspension was centrifuged (10,000× *g*) and the pellet was suspended in lysis buffer, added dropwise to 10-fold excess of 8 M guanidine hydrochloride with vigorous stirring, and left under stirring for 8 h at +4 °C. The solution was dialyzed against 20 mM Tris-HCl buffer, pH 7.5, 2 mM MgCl_2_, 2 mM 2-mercaptoethanol, 100 mM NaCl for 16 h at +4 °C, clarified by centrifugation (17,000× *g*, 30 min), and the supernatant was applied to Ni-NTA Excel column (GE Lifesciences, Chicago, IL, USA) equilibrated with 20 mM Tris-HCl buffer, pH 7.5, 2 mM MgCl_2_, 2 mM 2-mercaptoethanol, 250 mM NaCl. Elution of the target proteins was performed by a step gradient of imidazole (20 mM, 50 mM, 200 mM, and 500 mM) in the same buffer; the purity of the Cav and CavE preparations eluted at 500 mM imidazole was more than 95%.

### 2.3. Preparation of Recoverin Forms

Recombinant myristoylated recoverin and recoverin C39D mutant were obtained as described previously [35,37,41]. The myristoylation rate was assessed using analytical high performance liquid chromatography (HPLC) [42] and was more than 95%. The concentration of recoverin forms was measured spectrophotometrically using a molar extinction coefficient at 280 nm, calculated according to Pace et al. [43]. For comparative analysis, the concentration of dRec was calculated per mole of recoverin monomer.

### 2.4. Disulfide Dimerization of Recoverin In Vitro and Purification of dRec

To obtain analytical amounts of dRec, recombinant myristoylated recoverin (5 mg/mL) was dialyzed overnight against Ca^2+^-containing buffer (10 mM Tris-HCl buffer, pH 7.5, 100 mM NaCl, 1 mM CaCl_2_) in the presence of 500 μM H_2_O_2_ at 4 °C, resulting in recoverin dimerization rate of approximately 60-75%, according to SDS-PAGE. The dimer was separated from the monomer by gel-filtration chromatography on Superose-12 10/30 FPLC column (GE Lifesciences, Chicago, IL, USA). The fractions containing dRec were collected, dialyzed against 10 mM Tris-HCl buffer, pH 7.5, 100 mM NaCl overnight at 4 °C, freeze-dried, and stored at −70 °C.

To monitor disulfide dimerization of recoverin in the presence of caveolin-1 fragments, recombinant myristoylated recoverin (50 µM) was incubated with Cav or CavE (100 µM) in 10 mM Tris pH 7.5, 100 mM NaCl for 10 min at 20 °C and the mixture was dialyzed against 10 mM Tris-HCl buffer, pH 7.5, 100 mM NaCl, 1 mM EGTA, 1 mM H_2_O_2_ for 24 h at 4 °C. To study the effect of zinc, recoverin (50 µM) was incubated in 10 mM Tris-HCl buffer, pH 7.5, 100 mM NaCl, containing 1 mM EGTA or 4-fold molar excess of zinc (200 µM ZnCl_2_) [40] for 10 min at 20 °C and dialyzed against the same buffer containing 1 mM H_2_O_2_ for 24 h at +4 °C.

### 2.5. Circular Dichroism Spectroscopy

Far-UV circular dichroism (CD) measurements for Cav and CavE were carried out using a J-810 spectropolarimeter (JASCO, Inc., Tokyo, Japan), equipped with a Peltier-controlled cell holder at 20 °C. A quartz cell with a path length of 1 mm was utilized; bandwidth was 2 nm, with an averaging time of 2 s and accumulation of 3. The cell compartment was purged with nitrogen. The buffer conditions were 10 mM H_3_BO_3_-KOH, 50 mM NaCl, and 20 µM DTT at pH 9.0. The borate buffer and low concentrations of sodium chloride and DTT were used to avoid interfering absorption of the buffer components in the far-UV region. The sample protein concentration was 5.3–5.8 µM. Buffer contribution was subtracted from the spectrum. Quantitative estimations of the secondary structure contents were made using the CDPro software package [44], which includes the algorithms SELCON3, CDSSTR, and CONTIN. SDP48 and SMP56 reference protein sets were used for these evaluations. The final secondary structure fractions represent averaged values.

### 2.6. Dynamic Light Scattering

Measurements were performed using a Zetasizer Nano ZS device (Malvern Instruments Ltd., Malvern, UK) at 25 °C in buffer, containing 20 mM Tris-HCl (pH 7.5), 100 mM NaCl, and 1 mM DTT. The sample protein concentration was 200 µM. The accumulation time of the autocorrelation function for one measurement was 200 s. The refractive index (n_D_) and dynamic viscosity (η) of the buffer were set at n_D_ = 1.3338 and η = 0.95 mPa/s, respectively.

### 2.7. Pull-Down Assay

For analytical affinity chromatography (pull-down assay), Cav and CavE were covalently attached to Sepharose CL-4B [45]. Briefly, the resin was washed with distilled water and equilibrated with 0.2 M NaHCO_3_. BrCN dissolved in acetonitrile was added dropwise upon stirring, at the rate of 0.2 g of BrCN per 1 g of resin. The mixture was incubated at 15 °C for 5 min (pH 11). The resin was washed with 0.1 M HCl and incubated with Cav or CavE (1 mg/mL, 5 mg of protein per 1 g of resin) for 2 h at room temperature in 0.1 M NaHCO_3_ buffer, containing 0.5 M NaCl (pH 8.5). The remaining active groups were blocked by overnight incubation of the resin with 1 M ethanolamine solution in 0.1 M NaHCO_3_ buffer (pH 8.5) at 4 °C.

For pull-down assay, 50 μg of each recoverin form were incubated with 30 μL of 75% (*v*/*v*) suspension of Cav- or CavE-containing resin for 30 min at room temperature using thermomixer (Eppendorf) at 1200 rpm, in 20 mM Tris-HCl buffer (pH 7.5), 100 mM NaCl, 2 mM MgCl_2_, in the presence of 1 mM CaCl_2_ or 2 mM EGTA. After the incubation, the resin was washed three times with the corresponding buffer supplemented with 0.1% Tween-20. The bound proteins were eluted using SDS-PAGE sample buffer and analyzed by SDS-PAGE.

### 2.8. Surface Plasmon Resonance Spectroscopy

Surface plasmon resonance (SPR) measurements were carried out at 25 °C using the Bio-Rad ProteOn™ XPR36 system and ProteOn GLH sensor chip (Bio-Rad, Hercules, CA, USA) as previously described [14]. Ligand (40 μg/mL Cav or CavE in 10 mM sodium acetate, pH 4.0 buffer) was immobilized on the chip surface (up to 14,000 resonance units, RUs) by amine coupling, according to the manufacturer’s instructions. The remaining activated amine groups on the chip surface were blocked by 1 M ethanolamine solution. Analyte (2.5 μM to 30 μM Rec, dRec, or Rec-C39D) in a running buffer (10 mM HEPES buffer, pH 7.4, 150 mM NaCl, 2 mM DTT (except when dRec was used as the analyte), 0.05% TWEEN 20, and 1 mM CaCl_2_ or 1 mM EDTA) was supplied over the chip at the rate of 30 μL/min for 350 s, followed by flushing the chip with the running buffer for 1600–2400 s. The double-referenced SPR sensorgrams were globally fitted according to a heterogeneous ligand model. The equilibrium dissociation constants, KD, were evaluated using Bio-Rad ProteOn Manager™ v.3.1 software (Bio-Rad, Hercules, CA, USA). The sensor chip surface was regenerated by the passage of 0.5% SDS water solution for 50 s.

### 2.9. Cell Culture

MDCK (CCL-34; ATCC) and MDCK-Rec (see below) cell lines culturing was performed in Dulbecco’s modified Eagle’s medium (DMEM) supplemented with 10% fetal bovine serum (FBS), 2 mM L-glutamine, 4.5 g/L D-glucose, and penicillin/streptomycin at 37 °C in a humidified atmosphere containing 5% CO_2_. To prepare the MDCK cell line with stable expression of recoverin (MDCK-Rec), pCIneo-Rec plasmid was linearized by restriction at the BamH1 site. MDCK cells (1 × 10^7^ cells/mL) were electroporated on ice in 0.5 mL PBS with 10 μg pCIneo-Rec as a carrier at 450 V and 500 mF in a Gene Pulser unit (Bio-Rad). Cells were seeded in a 96-well plate (DMEM, 20% FBS). After 3 days, the selective antibiotic G418 was added to the medium at a concentration of 1 mg/mL. After 2–3 weeks of selection, individual clones were propagated for further analysis. Expression of recoverin was monitored by Western blotting (for the procedure, see below) of cell lysates, obtained by the homogenization of the cellular pellets from each well (approximately 30,000 cells) in 140 mM Tris-HCl buffer, pH 6.8, 2.2% SDS, 10 mM iodoacetamide.

For short-term treatments, H_2_O_2_ (3, 10, or 20 mM), vanadate (0.5 mM), and/or BAPTA-AM (5 μM) were added directly to the medium and incubated for the indicated periods.

### 2.10. Western Blotting

Western blotting of the cell lysates was performed as described elsewhere [46]. The staining of the blots was conducted using rabbit polyclonal antibodies against recoverin obtained in the previous study [47]. The content of dRec was monitored by performing SDS-PAGE under non-reducing conditions. Protein bands were visualized employing the Enhanced Chemiluminescence reagent kit and ChemiDoc™ XRS+ gel documentation system (Bio-Rad, Hercules, CA, USA). For quantitative assessments, the loading samples were normalized by total protein content (10 μg of total protein per track) and the amounts of recoverin forms were calculated by densitometric analysis of the bands using GelAnalyzer.2010a software (Istvan Lazar Jr., University of Budapest, Budapest, Hungary).

### 2.11. Immunocytochemistry and Microscopy

MDCK-Rec cells were grown on glass coverslips in six-well plates and treated as described above. All subsequent incubations were performed at room temperature using a plate shaker unless otherwise specified. For fixation, cells were washed with PBS (here and further without Mg^2+^ and Ca^2+^), incubated with 4% paraformaldehyde in PBS for 10 min, and washed with ice-cold PBS three times for 5 min. After that, cells were permeabilized by incubation with 0.2% Tween 20 in PBS for 20 min, washed three times with PBS, and blocked by incubation with 1% BSA, 22.52 mg/mL glycine in PBS with 0.1% Tween 20 (PBST) for 30 min. Caveolin-1/phosphorylated caveolin-1 and recoverin staining was performed by incubation of cells simultaneously with rabbit monoclonal antibodies against caveolin-1/phosphorylated caveolin-1 and mouse polyclonal antibodies against recoverin in 1% BSA in PBST overnight at 4 °C, with subsequent three times washing in PBST. Next, simultaneous incubation with goat anti-rabbit Alexa Fluor 555-conjugated IgG and goat anti-mouse Alexa Fluor 488-conjugated IgG was performed in 1% BSA in PBST for 1 h in the dark, with subsequent three times washing in PBST. Finally, cells were subjected to nuclear staining by incubation with 1 μg/mL DAPI in the dark for 1 min and subsequently washed with PBS. Coverslips with stained cells were mounted to a glass slide with a drop of Mowiol mounting medium, and allowed to solidify overnight at room temperature in the dark. Microscopy analysis of slides was performed with an upright LSM 880 microscope with Airyscan equipped with an Axiocam 506 mono camera using Zen Black 3.1 software (Zeiss, Oberkochen, Germany).

### 2.12. Data Analysis

Statistical data analysis was performed in SigmaPlot 11 software (Systat software, Chicago, IL, USA). Comparative analysis of results was performed using an unpaired two-tailed *t*-test.

## 3. Results

### 3.1. Preparation and Characterization of Oxidative Stress-Related Forms of Recoverin and Caveolin-1

To explore if modifications of recoverin and caveolin-1 associated with oxidative stress can affect their interaction, we first obtained and characterized recombinant proteins corresponding to their modified forms. In the case of recoverin, we prepared its reduced monomer (Rec), disulfide dimer (dRec), and C39D mutant (Rec-C39D) mimicking thiol oxidation of cysteine-39 to a sulfenic/sulfinic/sulfonic group. A detailed structural and functional analysis of these forms was performed in our previous studies [34,35,37]. Caveolin-1 is known to interact with recoverin via CSD (82–101 aa), a part of the oligomerization domain (61–101 aa) [14], whereas its oxidative stress-induced phosphorylation occurs at Y14 located in the N-terminal domain (1-81 aa) [4,8,9]. Therefore, for our purposes, we prepared a soluble fusion protein consisting of these two domains (1–101 aa) with (CavE) or without (Cav) Y14E substitution mimicking phosphorylation at Y14 [30,48] (Figure 1).

**Figure 2:**
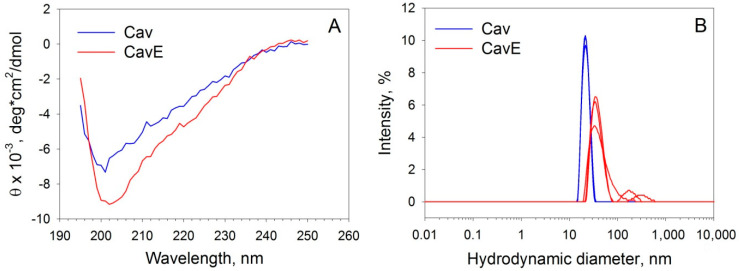
Secondary structure and oligomerization of caveolin-1 fragments. (**A**) Representative far-UV CD spectra for 5.5 µM Cav and CavE in 10 mM H_3_BO_3_-KOH buffer (pH 9.0), 50 mM NaCl, 20 µM DTT at 20 °C. (**B**) Hydrodynamic diameter of 200 µM Cav and CavE assessed by DLS in 20 mM Tris-HCl buffer (pH 7.5), 100 mM NaCl, 1 mM DTT at 25 °C. The data were determined in three independent measurements.

To investigate whether Y14E substitution affects specific structural features of the N-terminal region of caveolin-1, the molecular properties of Cav and CavE were examined using CD and dynamic light scattering (DLS) spectroscopies. According to CD data (Figure 2A, Table 1), Cav mainly consists of a random coil (approximately 40%) and β-folded layers (30%), which generally corresponds to the previous estimates for the analogous fragments of the protein [10,49]. Notably, CavE exhibited a similar secondary structure (Figure 2A, Table 1). The results of DLS experiments indicated that both fragments formed homogenous oligomers as evidenced by their hydrodynamic radii with narrow distribution (Figure 2B). Meanwhile, CavE formed larger particles as compared to Cav, in agreement with observations made for the analogous full-length proteins [50]. Overall, the recombinant protein samples represented feasible mimics of the signaling forms of caveolin-1 that were recognized as suitable for examination of the interaction of the scaffold protein with reduced and oxidized forms of recoverin.

### 3.2. Effect of Oxidative Stress-Associated Modifications of Recoverin and Caveolin-1 on Their Interaction In Vitro

**Figure 3:**
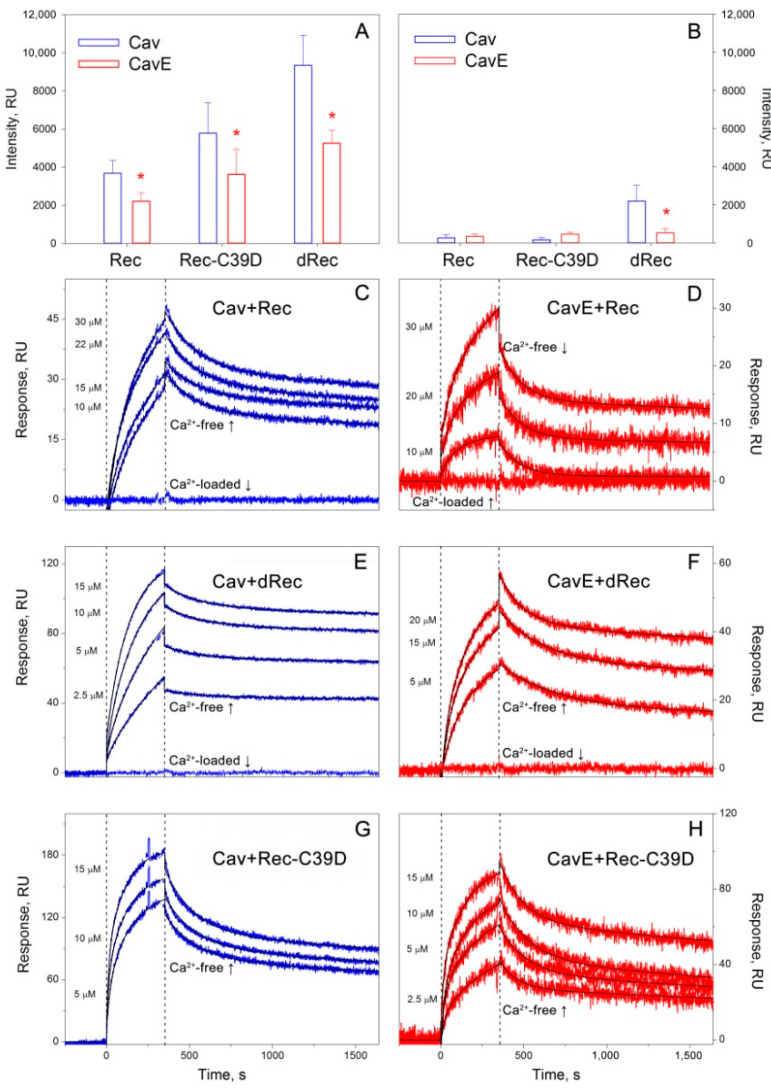
Interaction of reduced monomer (Rec), thiol oxidation mimicking mutant (Rec-C39D), or disulfide dimer (dRec) of recoverin with caveolin-1 fragments. (**A**,**B**) The results of pull-down assay of Rec, Rec-C39D, or dRec (1.7 mg/mL) with affinity resins containing immobilized Cav or CavE in 20 mM Tris-HCl buffer (pH 7.5), 100 mM NaCl, 2 mM MgCl_2_, in the absence (**A**) or the presence (**B**) of calcium (1 mM CaCl_2_ or 2 mM EGTA) at 25 °C. Error bars represent the weight fractions of the bound recoverin forms (in relative units, RU) determined from at least three independent experiments. *—*p* < 0.05. (**C**–**H**) Kinetics of the interaction between Rec (**C**,**D**), dRec (**E**,**F**), or Rec-C39D (**G**,**H**) (2.5 μM to 30 μM) with immobilized Cav or CavE in 10 mM HEPES buffer (pH 7.4), 150 mM NaCl, 2 mM DTT (except for dRec studies), 0.05% TWEEN20 in the absence (“Ca^2+^-free”) or in the presence (“Ca^2+^-loaded”) of calcium (1 mM CaCl_2_ or 1 mM EDTA), determined by SPR spectroscopy at 25 °C. Blue and red sensorgrams represent experimental data, while black curves are theoretical fits calculated according to the “heterogeneous ligand” model.

The interaction between the obtained recoverin and caveolin-1 forms was analyzed using two alternative approaches, namely pull-down assay, and SPR spectroscopy. The first approach involved co-precipitation of Rec, dRec, or Rec-C39D with specific resins obtained by covalent attachment of Cav or CavE to BrCN-activated Sepharose. In all cases, the interaction was dramatically suppressed in the presence of calcium (Figure 3A,B). In the case of caveolin-1 carrying phosphorylation-mimicking Y14E mutation (CavE), the affinity to intact recoverin was reduced by approximately 30–40%. Meanwhile, in the case of Rec-C39D and dRec, the interaction with Cav conversely enhanced 2-3-fold, with the most pronounced effect observed in the case of dRec. Notably, disulfide dimerization of recoverin adjusted the affinity of the protein to CavE to the level exceeding those observed for its complex with Cav, thereby overwhelming negative effects introduced by phosphorylation-mimicking Y14E mutation.

The same tendencies were observed in SPR spectroscopy studies allowing estimating of kinetic and equilibrium characteristics of the complexes. In these experiments, we monitored the interaction of Rec, dRec, or Rec-C39D supplied in the mobile phase with Cav or CavE immobilized on an SPR chip by amine coupling (Figure 3C–F). It was found that in the absence of calcium recoverin bound CavE 2–3 times weaker than Cav, but this effect became completely reversed upon thiol oxidation of the former. For instance, the equilibrium dissociation constant calculated for the dRec–Cav complex was lower than those for Rec–Cav complex by one order of magnitude and reached a nanomolar level (Table 2). In general, the affinity of recoverin forms to Cav and CavE increased in the order Rec < Rec-C39D < dRec in full accordance with the results of the pull-down assay. Similarly to pull-down experiments, in the presence of calcium, the binding of all forms was partially (Rec-C39D) or completely (Rec, dRec) suppressed.

We concluded that oxidative stress-associated modifications of recoverin and caveolin-1 produce significant but oppositely directed effects on the interaction of these proteins suggesting the existence of redox regulation of their signaling complex.

### 3.3. Effect of Caveolin-1 and Zinc on Disulfide Dimerization of Recoverin In Vitro

**Figure 4:**
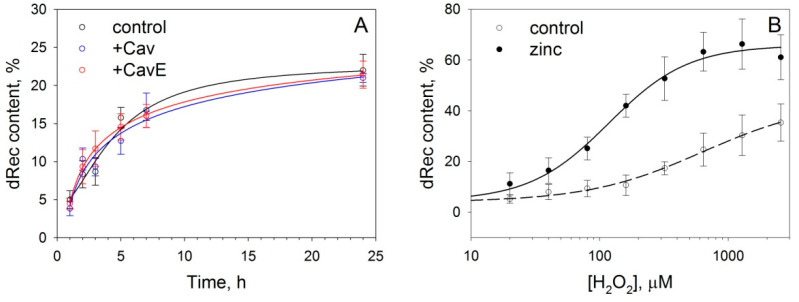
Disulfide dimerization of recoverin in vitro. (**A**) Kinetics of dRec formation during dialysis of 50 µM reduced recoverin against 10 mM Tris-HCl buffer (pH 7.5), 100 mM NaCl, 1 mM EGTA, containing 1 mM H_2_O_2_ for 24 h at 4 °C, with or without (control) preincubation in the presence of 100 µM Cav or 100 µM CavE. (**B**) Disulfide dimerization of 50 µM reduced recoverin during dialysis (24 h, 4 °C) against 10 mM Tris-HCl buffer (pH 7.5), 100 mM NaCl, containing indicated concentrations of H_2_O_2_ in the presence of 1 mM EGTA (“control”) or 4-fold molar excess of ZnCl_2_ (“zinc”). Weight fractions of dRec were determined by densitometric analysis of SDS-PAGE data from at least three independent experiments and plotted against time (**A**) or H_2_O_2_ concentrations (**B**).

Generally, the interaction of signaling proteins with caveolin-1 can modulate their functional properties and this modulation can be controlled via tyrosine (Y14) phosphorylation. Since disulfide dimerization of recoverin both affected the signaling activity of the protein [34,35] and promoted its binding to caveolin-1, we suggested that such oxidative modification can be stimulated by the interaction of recoverin with the scaffold and regulated by its phosphorylation. To test this suggestion, we monitored the kinetics of disulfide dimerization of recoverin in the presence of Cav or CavE in a Ca^2+^-free medium, i.e., under conditions promoting the formation of the recoverin–caveolin-1 complex (Figure 4A). It was demonstrated that neither of these forms affected dRec accumulation, regardless of the presence of calcium. Thus, it can be suggested that modifications of caveolin-1 and recoverin under oxidative stress conditions are non-interfering processes.

The oxidative stress-associated phosphorylation of caveolin-1 is mediated by Src kinase, which can be activated by zinc [51], the well-known mediator of redox homeostasis. Our preliminary experiments indicated that zinc can also affect thiol oxidation of Ca^2+^-loaded recoverin (unpublished data). Recently, we have demonstrated that zinc can stimulate non-covalent dimerization of recoverin—the first stage of dRec formation [40], but only in the case of a Ca^2+^-free conformer of the protein. Based on these data, we next verified if zinc can affect disulfide dimerization of recoverin in the absence of calcium, i.e., under conditions favoring the formation of recoverin–caveolin-1 complex. The experiments were performed using a 4-fold molar excess of zinc, which is necessary for the full saturation of the protein without significant effects on its thermal stability [40]. It was found that apo-recoverin is almost unsusceptible to disulfide dimerization, whereas the presence of zinc markedly promoted this reaction, apparently by decreasing the redox potential of dRec/Rec pair (Figure 4B).

In the aggregate, these findings, together with the literature, suggest that oxidative stress-associated modifications of recoverin and caveolin-1 may occur independently from each other, but both of them can be stimulated by free zinc.

### 3.4. Disulfide Dimerization of Recoverin and Phosphorylation of Caveolin-1 in Living Cells during Oxidative Stress

As it follows from our in vitro studies, phosphorylation of caveolin-1 and disulfide dimerization of recoverin produce significant effects on the interaction between the proteins. To test if these effects are physiologically relevant, we analyzed the feasibility of the abovementioned modifications in living cells in response to oxidative stress. The experiments were performed using a newly created model representing MDCK cells with stable expression of recoverin (MDCK-Rec cell line). The MDCK cell line was chosen as it is characterized by high endogenous expression of caveolin-1 [52]. The necessary condition for the redox regulation of the signaling complex between caveolin-1 and recoverin is the simultaneous formation of the corresponding forms of both proteins within the cell. Therefore, we first monitored the generation of phosphorylated caveolin-1 and dRec in response to oxidative stress, which was induced by exposure of the cells to increasing concentrations of hydrogen peroxide in the presence of vanadate [53].

**Figure 5:**
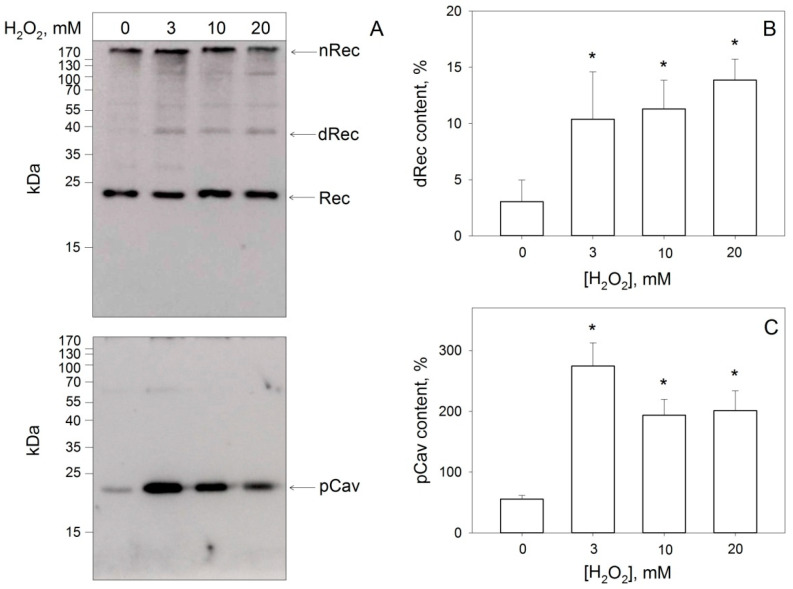
Disulfide dimerization of recoverin and tyrosine (Y14) phosphorylation of caveolin-1 in MDCK-Rec cells under oxidative stress conditions. (**A**) Cells were incubated with indicated concentrations of H_2_O_2_ in the presence of 0.5 mM vanadate for 10 min and their lysates were subjected to non-reducing Western blotting using antibodies against recoverin (upper panel) or P-caveolin-1 (lower panel). The positions of monomer (“Rec”), disulfide dimer (“dRec”), and disulfide aggregates (“nRec”) of recoverin, as well as P-caveolin-1 (“pCav”) are indicated by arrows. (**B**,**C**) Weight fractions of dRec (**B**) and pCav (**C**) estimated from Western blotting data from at least three independent experiments. *—*p* < 0.05 as compared to the data obtained for untreated cells.

Western blotting of the cellular lysates revealed that although a small fraction of Y14-phosphorylated caveolin-1 (P-caveolin-1) was present in the intact cells, its pronounced accumulation (up to 4-fold) occurred only in response to oxidative stress (Figure 5A,C). Importantly, under these conditions, we registered the simultaneous generation of oxidized forms of recoverin, including dRec and its aggregates (Figure 5A). The total fraction of the dimer was up to 10%, whereas the dRec:Rec ratio reached approximately 1:6 (Figure 5B). Overall, these findings confirmed that oxidative stress triggers Y14 phosphorylation of caveolin-1 in living cells and this process is accompanied by disulfide dimerization of recoverin.

### 3.5. Localization of Recoverin and Phosphorylated/Unphosphorylated Caveolin-1 during Oxidative Stress in Living Cells

**Figure 6:**
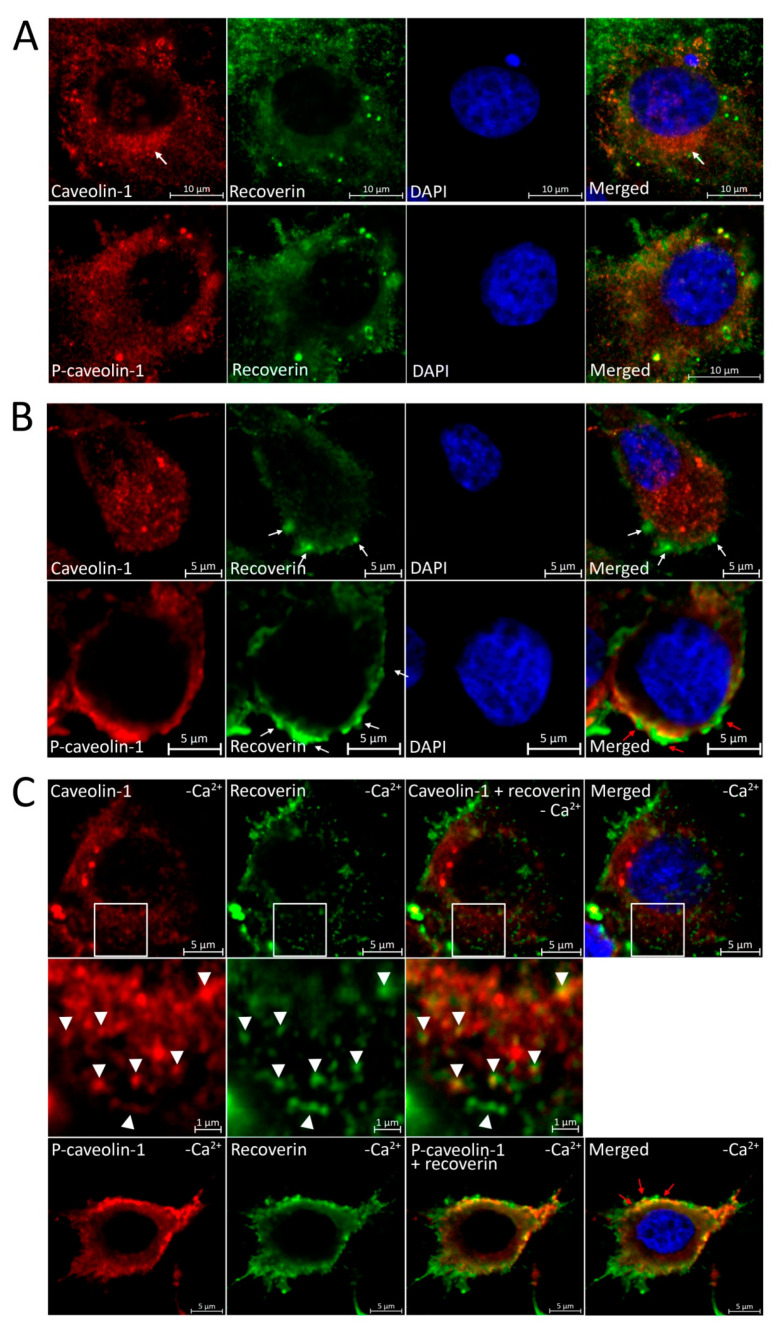
Localization of caveolin-1, P-caveolin-1, and recoverin in MDCK-Rec cells under oxidative stress conditions. Caveolin-1 and P-caveolin-1 are visualized by immunocytochemical analysis using rabbit monoclonal antibodies and goat anti-rabbit Alexa Fluor 555-conjugated IgG (red). Recoverin is visualized using mouse polyclonal antibodies and goat anti-mouse Alexa Fluor 488-conjugated IgG (green). Cell nuclei are stained with DAPI (blue). (**A**) Normal conditions. White arrow indicates the area of caveolin-1 localization in the Golgi complex. (**B**) Oxidative stress conditions (10 mM H_2_O_2_). White arrows indicate recruitment of recoverin to the plasma membrane. Red arrows point to the sites of co-localization of recoverin with P-caveolin-1. (**C**) Oxidative stress against the background of the calcium depletion conditions (5 μM BAPTA-AM). Red arrows point to the sites of co-localization of recoverin with P-caveolin-1. The insets with higher magnification (in the middle) demonstrate areas in the cytoplasm with co-localization of recoverin and non-phosphorylated caveolin-1 (indicated by white arrowheads).

The interaction between recoverin and caveolin-1 within a cell would manifest as their co-localization, which can be altered in oxidative stress due to the above-described modifications of the proteins. With this in mind, we examined the cellular localization of recoverin, caveolin-1, and P-caveolin-1 under normal and oxidative stress conditions by immunocytochemical analysis. In intact proliferating cells, recoverin and caveolin-1 demonstrated cytoplasmic localization with caveolin-1 clustering in the Golgi complex (Figure 6A). Upon H_2_O_2_ treatment, the P-caveolin-1 signal concentrated on the plasma membrane, compared to the still diffuse cytoplasmic distribution of unphosphorylated caveolin-1 (Figure 6B). Notably, the intensive recruitment of recoverin to the plasma membrane was also detected, resulting in its compartmentalization with P-caveolin-1. These alterations may reflect oxidative stress-promoted phosphorylation of caveolin-1 in the plasma membrane accompanied by disulfide dimerization of recoverin and its transfer into the high-affinity complex with caveolin-1/P-caveolin-1, in line with our biochemical data.

Since recoverin can bind plasma membrane directly via the myristoyl group exposed in the presence of calcium, the observed co-localization of the protein with P-caveolin-1 can reflect such interaction, as a free intracellular concentration of Ca^2+^ is known to increase during oxidative stress both in photoreceptor cells and MDCK cell line [54,55]. To consider this effect, the stress was induced against the background of the calcium depletion conditions, created by the treatment of the cells with BAPTA-AM, the specific chelator of Ca^2+^. Despite the inability of recoverin to directly interact with the phospholipid bilayer under these conditions, it still co-localized with phosphorylated caveolin-1 on the plasma membrane (Figure 6C, lower panel) suggesting a direct interaction between these proteins. Interestingly, we also noted the co-localization of recoverin with non-phosphorylated caveolin-1 in the cytoplasm (Figure 6C, middle panel), which agrees with the pronounced booster effect of the low calcium conditions toward the formation of recoverin–caveolin-1 complex.

Overall, the intracellular compartmentalization (and apparently interaction) of recoverin and caveolin-1 is altered under oxidative stress conditions and these alterations can be mediated by the phosphorylation state of caveolin-1 and the Ca^2+^-binding/redox state of recoverin.

## 4. Discussion

Our results indicate that oxidative stress induces two associated events, namely tyrosine (Y14) phosphorylation of caveolin-1 in the plasma membrane and accumulation of recoverin in the same regions of the membrane. These observations were made using MDCK-Rec cells exhibiting high expression of both proteins. Given that recoverin is specific for retinal photoreceptors, which are characterized by the high content of caveolin-1 and undergo oxidative damage in AMD [5,21,22,23], the employed cell line can be regarded as a model of AMD-related conditions. The increased redox potential of the cellular medium under stress conditions is known to induce the oxidation of Zn^2+^-binding proteins and elevation of free Zn^2+^ concentration [56,57]. Together these factors can favor the oxidation of Rec with the formation of dRec (see Figure 4B). According to our biophysical studies, dRec demonstrated relatively high (nanomolar) affinity to caveolin-1, even if the latter is phosphorylated (see Table 2). Thus, the observed accumulation of recoverin signal on the membrane likely reflects the formation of the complex between dRec and caveolin-1/P-caveolin-1. The complex was formed even upon depletion of intracellular calcium (see Figure 6C), i.e., under conditions that reduce membrane affinity of myristoylated recoverin but favor its interaction with caveolin-1 [14]. These observations suggest that caveolin-1 can mediate the localization of oxidized Ca^2+^-free recoverin on the plasma membrane. In the cytoplasm, the co-localization of recoverin and caveolin-1 was less evident, apparently due to the much lower local concentration of caveolin-1, which could be present in membranes of intracellular vesicles and organelles, such as endoplasmatic reticulum and Golgi complex [58,59]. Accordingly, the focal signs of the recoverin–caveolin-1 complex in the cytoplasm became detectable only upon the oxidation of recoverin and the loss of calcium by this protein (Figure 6C), i.e., under conditions significantly enhancing its affinity to caveolin-1 and, correspondingly, increasing the amount of the complex.

**Figure 7:**
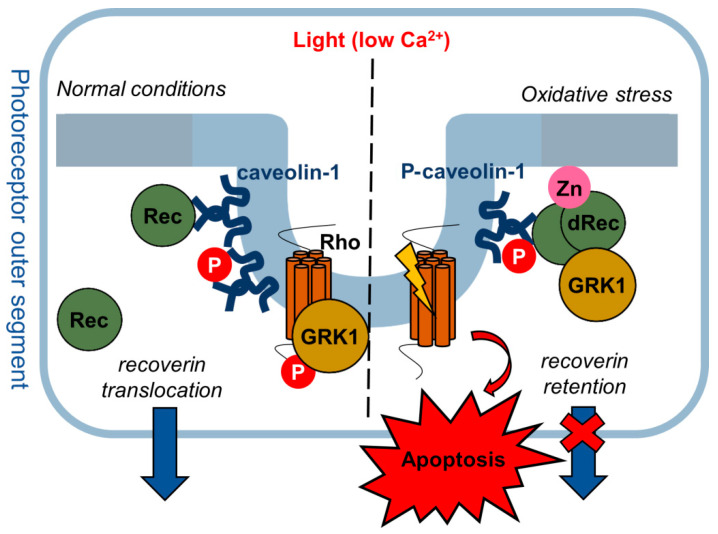
The hypothetical function of caveolin-1 complex with Rec/dRec in photoreceptors. Under normal light conditions (low calcium), recoverin forms a complex with caveolin-1 in DRMs of OS, which can be attenuated by tyrosine (Y14) phosphorylation, enabling translocation of recoverin to IS. In oxidative stress, the increased zinc concentration induces the formation of dRec, which retains in OS due to increased affinity to P-caveolin-1. The ability of dRec to constitutively inhibit rhodopsin kinase (GRK1) can slow down rhodopsin desensitization, promote oxidative stress, and induce apoptosis.

Tyrosine (Y14) phosphorylation of caveolin-1 represents a functional switch regulating the interaction of the scaffold protein with signaling partners in various cell types [60,61,62]. Our in vitro data demonstrated that the interaction between caveolin-1 and recoverin can be regulated by such phosphorylation, which diminishes the affinity of their signaling complex. Notably, in recoverin-expressing photoreceptor cells of the mature retina, caveolin-1 and P-caveolin-1 display different localization: the former is present in IS and cell bodies, whereas the latter is accumulated in OS [31]. Given the light-induced decrease in intracellular calcium and the associated translocation of recoverin from OS to IS (see Section 1), we can suggest that under normal conditions, a considerable part of recoverin will be bound to non-phosphorylated caveolin-1 in the IS. Yet, the retina is highly vulnerable to photo-oxidative stress, a crucial factor in AMD pathogenesis [22]. The stress will trigger the formation of dRec [34,35], which might be accelerated by an increased concentration of free zinc (see Figure 4B) characteristic of light-adapted photoreceptors [63]. These conditions might disturb the well-known mechanism of light-induced translocation of recoverin in photoreceptors [19] by retaining it in OS due to the increased affinity of dRec and other oxidized forms of the protein to P-caveolin-1. The ability of dRec to constitutively inhibit GRK1 in OS might slow down rhodopsin desensitization in the light, thereby further promoting oxidative stress and inducing apoptosis of photoreceptors (Figure 7), the major driving forces of AMD [5,35].

In general, the increased affinity of oxidized recoverin to caveolin-1 could be related to the segregation function of the caveolae [64]. Indeed, oxidized monomer (C39D mutant) of recoverin and dRec are characterized by altered function, lower structural stability, and increased susceptibility to aggregation [34,35,37] and accumulation of these aberrant forms in photoreceptor caveolae/DRMs would segregate them from the reduced protein. Caveolin-1 is permanently utilized by the formation of 8S caveolar complexes followed by ubiquitination and proteasomal degradation [65]. Thus, the selective recognition and labeling of the oxidized forms of recoverin via interaction with caveolin-1 can mediate their recycling in the cell. Even so, the overload with these proteins of caveolae/DRMs of OS can trigger the rapid implementation of the abovementioned mechanism of photoreceptor apoptosis, promoting the development of AMD.

It should be noted that two factors regulating the recoverin–caveolin-1 complex, namely oxidative stress, and alterations in zinc concentration, are linked to each other and represent crucial elements of AMD pathogenesis [66,67]. Although the exact role of zinc in AMD is controversial [68], in general, this disease is associated with zinc deficiency, which deteriorates responses of RPE and photoreceptor cells to oxidative stress, since zinc contributes to the antioxidant defense by being a cofactor of superoxide dismutase (SOD) and upregulating other antioxidant proteins, including metallothioneins (for review, see [67]). Consistently, zinc supplementation protects photoreceptors from light-induced degeneration [27] and stimulates the phagolysosomal activity of RPE towards phototoxic lipofuscin, which is dramatically reduced in AMD [69]. Notably, the phagolysosomal activity of RPE cells is regulated by caveolin-1 [70]. Moreover, genetic depletion of this protein promotes choroidal and retinal neovascularization, a hallmark of wet AMD [71]. Consistently, caveolin-1 was shown to be downregulated in the Bruch membrane/choroid complex of individuals with AMD [72]. Meanwhile, in the RPE cells of AMD patients, expression of caveolin-1 is significantly increased [73] and promotes cellular senescence, thereby contributing to the progression of geographic atrophy in dry AMD [74]. In photoreceptors, AMD-associated alterations in caveolin-1 expression remain so far unspecified, but in the case of a similar increase, it might promote oxidative stress-induced trapping of dRec in OS, thereby exacerbating light-induced apoptosis of these cells.

Of particular interest are the structural aspects underlying the observed decrease in the affinity of phosphorylated caveolin-1 to wild-type recoverin and the significant increase in affinity of oxidized monomer/C39D mutant and dRec to wild-type caveolin-1. The binding of recoverin and tyrosine (Y14) phosphorylation involves different parts of caveolin-1, namely the SD and the N-terminal domain of the protein. Since a significant part of the latter residues (1–48 aa) remains unresolved so far [4], the relative positions of these domains within the protein remain unclear. Previous studies demonstrate that Y14 is involved in caveolin-1 oligomerization and its phosphorylation induces conformational changes destabilizing the caveolar complex [50]. The resolved part of the N-terminal domain (the so-called “pin motif”, 49–60 aa) interacts with the SD of the second neighboring protomer: Arg54 intercalates into a pocket formed by His79 and Trp85. Moreover, Arg54 interacts with E74 from the N-terminal domain of the third caveolin-1 molecule [4]. Thus, we can speculate that the negative charge gained by the phosphorylation (or Y-to-E substitution) may perturb these interactions, leading to the abovementioned conformational changes and thereby altering the target recognition properties of SD. The same interactions and similar effects may be realized in the case of the Cav (1–101 aa) fragment, employed in this study. Indeed, similarly to full-length caveolin-1 [4], Cav forms circle structures, although they contained seven copies of the construct [49] instead of eleven in the full-length protein. These “bundles” were shown to form high molecular weight aggregates [49], which agrees with the results of our DLS studies (Figure 2B). The structural perturbations induced by Y-to-E substitution may destabilize these bundles, thereby promoting caveolin-1 aggregation, in agreement with our DLS data (Figure 2B).

The enhanced binding of dRec to wild-type caveolin-1 may be related to the increased hydrophobicity of the dimer [35], which could reflect enhanced accessibility of the hydrophobic residues of the caveolin-1-binding site [14]. Moreover, Cav (1–101 aa) fragment may exist as a dimer [75] or form dimeric units within the oligomeric complexes [49], where two copies of SD are approached each other (the architecture of 8S caveolin-1 complex supports this suggestion [4]). This configuration makes the dimeric form of recoverin (dRec) potentially more preferable for the interaction as compared to the monomer. In the case of the C39D mutant (oxidized monomer) of recoverin, the enhanced caveolin-1 binding can be related to altered calcium-binding properties of the protein. Indeed, the global structure of C39D displayed no obvious differences from that of WT recoverin, but the mutant exhibited approximately 10-fold reduced calcium affinity [36]. Thus, the probability of formation of the calcium-free conformer, which is intended for caveolin-1 binding [14], in the case of the C39D mutant is significantly increased. Further structural studies are required for establishing the detailed mechanisms underlying redox regulation of the signaling complex between caveolin-1 and recoverin.

## Figures and Tables

**Table 1:** Secondary structure contents and hydrodynamic diameter (d*_h_*)/molecular weight (M*_r_*) values determined for Cav and CavE from CD and DLS data in Figure 1.

Caveolin-1 Form	CD				DLS	
	α-Helix,%	β-Sheet, %	Turn, %	Random Coil, %	d*_h_*, nm	M*_r_*, kDa
Cav	7.2 ± 3.7	30.1 ± 5.6	19.8 ± 3.1	40.3 ± 8.3	21.4 ± 4.6	866 ± 206
CavE	7.4 ± 2.9	26.2 ± 5.2	21.1 ± 3.8	44.7 ± 9.1	35.1 ± 13.6	2700 ± 1400

**Table 2:** Parameters of the heterogeneous ligand model, describing the SPR data on the kinetics of interaction between reduced monomer (Rec), disulfide dimer (dRec), or thiol oxidation mimicking mutant (Rec-C39D) of recoverin with caveolin-1 fragments at 25 °C.

Recoverin Form	Cav K_D_, nM (k*_off_*, s^−1^)		CavE K_D_, nM (k*_off_*, s^−1^)	
	2 mM Ca^2+^	2 mM EGTA	2 mM Ca^2+^	2 mM EGTA
Rec	n/d ^1^	490 ± 160 ((1.05 ± 0.18) × 10^−4^)	n/d	1110 ± 66 ((8.81 ± 2.59) × 10^−5^)
dRec	n/d	48.6 ± 1.1 ((2.44 ± 0.59) × 10^−5^)	n/d	218 ± 87 ((8.08 ± 0.27) × 10^−5^)
Rec-C39D	376 ± 173 ((1.86 ± 0.13) × 10^−4^)	140 ± 44 ((1.17 ± 0.76) × 10^−4^)	629 ± 257 ((2.02 ± 0.21) × 10^−4^)	329 ± 145 ((1.72 ± 0.15) × 10^−4^)

^1^ No binding detected.

## Data Availability

Not applicable.

## References

[1] Cohen A.W., Hnasko R., Schubert W., Lisanti M.P. (2004). Role of caveolae and caveolins in health and disease. Physiol. Rev..

[2] Boscher C., Nabi I.R. (2012). Caveolin-1: Role in cell signaling. Adv. Exp. Med. Biol..

[3] Del Pozo M.A., Lolo F.N., Echarri A. (2021). Caveolae: Mechanosensing and mechanotransduction devices linking membrane trafficking to mechanoadaptation. Curr. Opin. Cell Biol..

[4] Porta J.C., Han B., Gulsevin A., Chung J.M., Peskova Y., Connolly S., McHaourab H.S., Meiler J., Karakas E., Kenworthy A.K. (2022). Molecular architecture of the human caveolin-1 complex. Sci. Adv..

[5] Gu X., Reagan A.M., McClellan M.E., Elliott M.H. (2017). Caveolins and caveolae in ocular physiology and pathophysiology. Prog. Retin. Eye Res..

[6] Kovtun O., Tillu V.A., Ariotti N., Parton R.G., Collins B.M. (2015). Cavin family proteins and the assembly of caveolae. J. Cell Sci..

[7] Byrne D.P., Dart C., Rigden D.J. (2012). Evaluating Caveolin Interactions: Do Proteins Interact with the Caveolin Scaffolding Domain through a Widespread Aromatic Residue-Rich Motif?. PLoS ONE.

[8] Root K.T., Plucinsky S.M., Glover K.J. (2015). Recent progress in the topology, structure, and oligomerization of caveolin: A building block of caveolae. Curr. Top. Membr..

[9] Wong T.H., Dickson F.H., Timmins L.R., Nabi I.R. (2020). Tyrosine phosphorylation of tumor cell caveolin-1: Impact on cancer progression. Cancer Metastasis Rev..

[10] Hoop C.L., Sivanandam V.N., Kodali R., Srnec M.N., van der Wel P.C. (2012). Structural characterization of the caveolin scaffolding domain in association with cholesterol-rich membranes. Biochemistry.

[11] Plucinsky S.M., Glover K.J. (2015). Secondary Structure Analysis of a Functional Construct of Caveolin-1 Reveals a Long C-Terminal Helix. Biophys. J..

[12] Schlegel A., Arvan P., Lisanti M.P. (2001). Caveolin-1 binding to endoplasmic reticulum membranes and entry into the regulated secretory pathway are regulated by serine phosphorylation. Protein sorting at the level of the endoplasmic reticulum. J. Biol. Chem..

[13] Li X., McClellan M.E., Tanito M., Garteiser P., Towner R., Bissig D., Berkowitz B.A., Fliesler S.J., Woodruff M.L., Fain G.L. (2012). Loss of caveolin-1 impairs retinal function due to disturbance of subretinal microenvironment. J. Biol. Chem..

[14] Vladimirov V.I., Zernii E.Y., Baksheeva V.E., Wimberg H., Kazakov A.S., Tikhomirova N.K., Nemashkalova E.L., Mitkevich V.A., Zamyatnin A.A., Lipkin V.M. (2018). Photoreceptor calcium sensor proteins in detergent-resistant membrane rafts are regulated via binding to caveolin-1. Cell Calcium.

[15] Miyagawa Y., Ohguro H., Odagiri H., Maruyama I., Maeda T., Maeda A., Sasaki M., Nakazawa M. (2003). Aberrantly expressed recoverin is functionally associated with G-protein-coupled receptor kinases in cancer cell lines. Biochem. Biophys. Res. Commun..

[16] Zernii E.Y., Zinchenko D.V., Vladimirov V.I., Grigoriev I.I., Skorikova E.E., Baksheeva V.E., Lipkin V.M., Philippov P.P., Senin I.I. (2014). Ca^2+^-dependent regulatory activity of recoverin in photoreceptor raft structures: The role of caveolin-1. Biochem. (Mosc.) Suppl. Ser. A Membr. Cell Biol..

[17] Philippov P.P., Zernii E.Y., Choi S. (2018). Recoverin. Encyclopedia of Signaling Molecules.

[18] Zang J., Neuhauss S.C.F. (2018). The Binding Properties and Physiological Functions of Recoverin. Front. Mol. Neurosci..

[19] Strissel K.J., Lishko P.V., Trieu L.H., Kennedy M.J., Hurley J.B., Arshavsky V.Y. (2005). Recoverin undergoes light-dependent intracellular translocation in rod photoreceptors. J. Biol. Chem..

[20] Makino C.L., Dodd R.L., Chen J., Burns M.E., Roca A., Simon M.I., Baylor D.A. (2004). Recoverin regulates light-dependent phosphodiesterase activity in retinal rods. J. Gen. Physiol..

[21] Baksheeva V.E., Tiulina V.V., Tikhomirova N.K., Gancharova O.S., Komarov S.V., Philippov P.P., Zamyatnin A.A., Senin I.I., Zernii E.Y. (2018). Suppression of Light-Induced Oxidative Stress in the Retina by Mitochondria-Targeted Antioxidant. Antioxidants.

[22] Organisciak D.T., Vaughan D.K. (2010). Retinal light damage: Mechanisms and protection. Prog. Retin. Eye Res..

[23] Fleckenstein M., Keenan T.D.L., Guymer R.H., Chakravarthy U., Schmitz-Valckenberg S., Klaver C.C., Wong W.T., Chew E.Y. (2021). Age-related macular degeneration. Nat. Rev. Dis. Prim..

[24] Grahn B.H., Paterson P.G., Gottschall-Pass K.T., Zhang Z. (2001). Zinc and the eye. J. Am. Coll. Nutr..

[25] Ugarte M., Osborne N.N. (2014). Recent advances in the understanding of the role of zinc in ocular tissues. Metallomics.

[26] Sheline C.T., Zhou Y., Bai S. (2010). Light-induced photoreceptor and RPE degeneration involve zinc toxicity and are attenuated by pyruvate, nicotinamide, or cyclic light. Mol. Vis..

[27] Organisciak D., Wong P., Rapp C., Darrow R., Ziesel A., Rangarajan R., Lang J. (2012). Light-induced retinal degeneration is prevented by zinc, a component in the age-related eye disease study formulation. Photochem. Photobiol..

[28] Sanguinetti A.R., Mastick C.C. (2003). c-Abl is required for oxidative stress-induced phosphorylation of caveolin-1 on tyrosine 14. Cell. Signal..

[29] Chen D.B., Li S.M., Qian X.X., Moon C., Zheng J. (2005). Tyrosine phosphorylation of caveolin 1 by oxidative stress is reversible and dependent on the c-src tyrosine kinase but not mitogen-activated protein kinase pathways in placental artery endothelial cells. Biol. Reprod..

[30] Wehinger S., Ortiz R., Diaz M.I., Aguirre A., Valenzuela M., Llanos P., Mc Master C., Leyton L., Quest A.F. (2015). Phosphorylation of caveolin-1 on tyrosine-14 induced by ROS enhances palmitate-induced death of beta-pancreatic cells. Biochim. Biophys. Acta.

[31] Berta A.I., Boesze-Battaglia K., Magyar A., Szel A., Kiss A.L. (2011). Localization of caveolin-1 and c-src in mature and differentiating photoreceptors: Raft proteins co-distribute with rhodopsin during development. J. Mol. Histol..

[32] Elliott M.H., Ghalayini A.J. (2008). Phosphorylation of caveolin-1 in bovine rod outer segments in vitro by an endogenous tyrosine kinase. Adv. Exp. Med. Biol..

[33] Permyakov S.E., Nazipova A.A., Denesyuk A.I., Bakunts A.G., Zinchenko D.V., Lipkin V.M., Uversky V.N., Permyakov E.A. (2007). Recoverin as a redox-sensitive protein. J. Proteome Res..

[34] Zernii E.Y., Nazipova A.A., Gancharova O.S., Kazakov A.S., Serebryakova M.V., Zinchenko D.V., Tikhomirova N.K., Senin I.I., Philippov P.P., Permyakov E.A. (2015). Light-induced disulfide dimerization of recoverin under ex vivo and in vivo conditions. Free. Radic. Biol. Med..

[35] Zernii E.Y., Nazipova A.A., Nemashkalova E.L., Kazakov A.S., Gancharova O.S., Serebryakova M.V., Tikhomirova N.K., Baksheeva V.E., Vladimirov V.I., Zinchenko D.V. (2018). Light-Induced Thiol Oxidation of Recoverin Affects Rhodopsin Desensitization. Front. Mol. Neurosci..

[36] Ranaghan M.J., Kumar R.P., Chakrabarti K.S., Buosi V., Kern D., Oprian D.D. (2013). A highly conserved cysteine of neuronal calcium-sensing proteins controls cooperative binding of Ca^2+^ to recoverin. J. Biol. Chem..

[37] Permyakov S.E., Zernii E.Y., Knyazeva E.L., Denesyuk A.I., Nazipova A.A., Kolpakova T.V., Zinchenko D.V., Philippov P.P., Permyakov E.A., Senin I.I. (2012). Oxidation mimicking substitution of conservative cysteine in recoverin suppresses its membrane association. Amino Acids.

[38] Permyakov S.E., Cherskaya A.M., Wasserman L.A., Khokhlova T.I., Senin I.I., Zargarov A.A., Zinchenko D.V., Zernii E.Y., Lipkin V.M., Philippov P.P. (2003). Recoverin is a zinc-binding protein. J. Proteome Res..

[39] Tsvetkov P.O., Roman A.Y., Baksheeva V.E., Nazipova A.A., Shevelyova M.P., Vladimirov V.I., Buyanova M.F., Zinchenko D.V., Zamyatnin A.A., Devred F. (2018). Functional Status of Neuronal Calcium Sensor-1 Is Modulated by Zinc Binding. Front. Mol. Neurosci..

[40] Baksheeva V.E., Tsvetkov P.O., Zalevsky A.O., Vladimirov V.I., Gorokhovets N.V., Zinchenko D.V., Permyakov S.E., Devred F., Zernii E.Y. (2022). Zinc Modulation of Neuronal Calcium Sensor Proteins: Three Modes of Interaction with Different Structural Outcomes. Biomolecules.

[41] Permyakov S.E., Vologzhannikova A.S., Nemashkalova E.L., Kazakov A.S., Denesyuk A.I., Denessiouk K., Baksheeva V.E., Zamyatnin A.A., Zernii E.Y., Uversky V.N. (2019). Experimental Insight into the Structural and Functional Roles of the ‘Black’ and ‘Gray’ Clusters in Recoverin, a Calcium Binding Protein with Four EF-Hand Motifs. Molecules.

[42] Vladimirov V.I., Baksheeva V.E., Mikhailova I.V., Ismailov R.G., Litus E.A., Tikhomirova N.K., Nazipova A.A., Permyakov S.E., Zernii E.Y., Zinchenko D.V. (2020). A Novel Approach to Bacterial Expression and Purification of Myristoylated Forms of Neuronal Calcium Sensor Proteins. Biomolecules.

[43] Pace C.N., Vajdos F., Fee L., Grimsley G., Gray T. (1995). How to measure and predict the molar absorption coefficient of a protein. Protein Sci..

[44] Sreerama N., Venyaminov S.Y., Woody R.W. (2000). Estimation of protein secondary structure from circular dichroism spectra: Inclusion of denatured proteins with native proteins in the analysis. Anal. Biochem..

[45] Kavran J.M., Leahy D.J. (2014). Coupling antibody to cyanogen bromide-activated sepharose. Methods Enzymol..

[46] Baksheeva V.E., Baldin A.V., Zalevsky A.O., Nazipova A.A., Kazakov A.S., Vladimirov V.I., Gorokhovets N.V., Devred F., Philippov P.P., Bazhin A.V. (2021). Disulfide Dimerization of Neuronal Calcium Sensor-1: Implications for Zinc and Redox Signaling. Int. J. Mol. Sci..

[47] Senin I.I., Tikhomirova N.K., Churumova V.A., Grigoriev I.I., Kolpakova T.A., Zinchenko D.V., Philippov P.P., Zernii E.Y. (2011). Amino acid sequences of two immune-dominant epitopes of recoverin are involved in Ca^2+^/recoverin-dependent inhibition of phosphorylation of rhodopsin. Biochemistry.

[48] Ortiz R., Diaz J., Diaz N., Lobos-Gonzalez L., Cardenas A., Contreras P., Diaz M.I., Otte E., Cooper-White J., Torres V. (2016). Extracellular matrix-specific Caveolin-1 phosphorylation on tyrosine 14 is linked to augmented melanoma metastasis but not tumorigenesis. Oncotarget.

[49] Fernandez I., Ying Y., Albanesi J., Anderson R.G. (2002). Mechanism of caveolin filament assembly. Proc. Natl. Acad. Sci. USA.

[50] Zimnicka A.M., Husain Y.S., Shajahan A.N., Sverdlov M., Chaga O., Chen Z., Toth P.T., Klomp J., Karginov A.V., Tiruppathi C. (2016). Src-dependent phosphorylation of caveolin-1 Tyr-14 promotes swelling and release of caveolae. Mol. Biol. Cell.

[51] Manzerra P., Behrens M.M., Canzoniero L.M., Wang X.Q., Heidinger V., Ichinose T., Yu S.P., Choi D.W. (2001). Zinc induces a Src family kinase-mediated up-regulation of NMDA receptor activity and excitotoxicity. Proc. Natl. Acad. Sci. USA.

[52] Vogel U., Sandvig K., van Deurs B. (1998). Expression of caveolin-1 and polarized formation of invaginated caveolae in Caco-2 and MDCK II cells. J. Cell Sci..

[53] Vepa S., Scribner W.M., Natarajan V. (1997). Activation of protein phosphorylation by oxidants in vascular endothelial cells: Identification of tyrosine phosphorylation of caveolin. Free Radic. Biol. Med..

[54] Castro J., Bittner C.X., Humeres A., Montecinos V.P., Vera J.C., Barros L.F. (2004). A cytosolic source of calcium unveiled by hydrogen peroxide with relevance for epithelial cell death. Cell Death Differ.

[55] Donovan M., Carmody R.J., Cotter T.G. (2001). Light-induced photoreceptor apoptosis in vivo requires neuronal nitric-oxide synthase and guanylate cyclase activity and is caspase-3-independent. J. Biol. Chem..

[56] Maret W., Vallee B.L. (1998). Thiolate ligands in metallothionein confer redox activity on zinc clusters. Proc. Natl. Acad. Sci. USA.

[57] Maret W., Krezel A. (2007). Cellular zinc and redox buffering capacity of metallothionein/thionein in health and disease. Mol. Med..

[58] Simon L., Campos A., Leyton L., Quest A.F.G. (2020). Caveolin-1 function at the plasma membrane and in intracellular compartments in cancer. Cancer Metastasis Rev..

[59] Li W.P., Liu P., Pilcher B.K., Anderson R.G. (2001). Cell-specific targeting of caveolin-1 to caveolae, secretory vesicles, cytoplasm or mitochondria. J. Cell Sci..

[60] Couet J., Sargiacomo M., Lisanti M.P. (1997). Interaction of a receptor tyrosine kinase, EGF-R, with caveolins. Caveolin binding negatively regulates tyrosine and serine/threonine kinase activities. J. Biol. Chem..

[61] Yamamoto M., Toya Y., Schwencke C., Lisanti M.P., Myers M.G., Ishikawa Y. (1998). Caveolin is an activator of insulin receptor signaling. J. Biol. Chem..

[62] Rathor N., Zhuang R., Wang J.Y., Donahue J.M., Turner D.J., Rao J.N. (2014). Src-mediated caveolin-1 phosphorylation regulates intestinal epithelial restitution by altering Ca(^2+^) influx after wounding. Am. J. Physiol. Gastrointest. Liver Physiol..

[63] Ugarte M., Osborne N.N. (1999). The localization of free zinc varies in rat photoreceptors during light and dark adaptation. Exp. Eye Res..

[64] Oh P., Schnitzer J.E. (2001). Segregation of heterotrimeric G proteins in cell surface microdomains. G(q) binds caveolin to concentrate in caveolae, whereas G(i) and G(s) target lipid rafts by default. Mol. Biol. Cell.

[65] Hayer A., Stoeber M., Ritz D., Engel S., Meyer H.H., Helenius A. (2010). Caveolin-1 is ubiquitinated and targeted to intralumenal vesicles in endolysosomes for degradation. J. Cell Biol..

[66] Beatty S., Koh H., Phil M., Henson D., Boulton M. (2000). The role of oxidative stress in the pathogenesis of age-related macular degeneration. Surv. Ophthalmol..

[67] Blasiak J., Pawlowska E., Chojnacki J., Szczepanska J., Chojnacki C., Kaarniranta K. (2020). Zinc and Autophagy in Age-Related Macular Degeneration. Int. J. Mol. Sci..

[68] Wood J.P., Osborne N.N. (2003). Zinc and energy requirements in induction of oxidative stress to retinal pigmented epithelial cells. Neurochem. Res..

[69] Julien S., Biesemeier A., Kokkinou D., Eibl O., Schraermeyer U. (2011). Zinc deficiency leads to lipofuscin accumulation in the retinal pigment epithelium of pigmented rats. PLoS ONE.

[70] Sethna S., Chamakkala T., Gu X., Thompson T.C., Cao G., Elliott M.H., Finnemann S.C. (2016). Regulation of Phagolysosomal Digestion by Caveolin-1 of the Retinal Pigment Epithelium Is Essential for Vision. J. Biol. Chem..

[71] Jiang Y., Lin X., Tang Z., Lee C., Tian G., Du Y., Yin X., Ren X., Huang L., Ye Z. (2017). Critical role of caveolin-1 in ocular neovascularization and multitargeted antiangiogenic effects of cavtratin via JNK. Proc. Natl. Acad. Sci. USA.

[72] Yuan X., Gu X., Crabb J.S., Yue X., Shadrach K., Hollyfield J.G., Crabb J.W. (2010). Quantitative proteomics: Comparison of the macular Bruch membrane/choroid complex from age-related macular degeneration and normal eyes. Mol. Cell. Proteom. MCP.

[73] Senabouth A., Daniszewski M., Lidgerwood G.E., Liang H.H., Hernandez D., Mirzaei M., Keenan S.N., Zhang R., Han X., Neavin D. (2022). Transcriptomic and proteomic retinal pigment epithelium signatures of age-related macular degeneration. Nat. Commun..

[74] Shimizu H., Yamada K., Suzumura A., Kataoka K., Takayama K., Sugimoto M., Terasaki H., Kaneko H. (2020). Caveolin-1 Promotes Cellular Senescence in Exchange for Blocking Subretinal Fibrosis in Age-Related Macular Degeneration. Investig. Ophthalmol. Vis. Sci..

[75] Kim J., Shin J., Park H. (2003). Structural characterization for N-terminal domain of caveolin-1. Korean J. Biol. Sci..

